# An affixed, ingestible blood-sensing monitor for continuous monitoring after high-risk endoscopic procedures: a case series

**DOI:** 10.1016/j.igie.2025.09.008

**Published:** 2025-09-11

**Authors:** Kimberly F. Schuster, Alexandra Goad, Steven N. Steinway

**Affiliations:** 1 Tufts University School of Medicine, Boston, Massachusetts, USA; 2 University of Louisville School of Medicine, Louisville, Kentucky, USA; 3 Division of Gastroenterology, Hepatology, and Endoscopy, Brigham and Women's Hospital, Boston, Massachusetts, USA

## Abstract

**Background and Aims:**

Detecting postprocedural bleeding (PPB) after high-risk endoscopic interventions remains challenging. A fixed intraluminal bleeding sensor could improve PPB detection.

**Methods:**

This case series included patients undergoing endoscopic retrograde cholangiopancreatography (ERCP) who were monitored for 24 to 48 hours by affixing a capsule-based bleeding sensor to the duodenal wall.

**Results:**

Five patients underwent ERCP with sphincterotomy (n = 3) or ampullectomy (n = 2). The sensor was positive in 3 and negative in 2. Repeat endoscopy confirmed bleeding in 2 sensor-positive cases and 1 sensor-negative case, and hemostasis was successfully achieved. Sensor migration occurred in 3 cases. One patient with persistently negative readings was safely discharged. No adverse events occurred over 3 to 6 months of follow-up.

**Conclusions:**

This pilot series supports the feasibility of a fixed capsule–based sensor for real-time PPB detection as an adjunct to standard monitoring. Larger studies are needed to validate effectiveness and optimize deployment.

## Introduction

Postprocedural bleeding (PPB) is an adverse event in 2% to 5% of endoscopic sphincterotomies (ESs) and up to 30% of ampullectomies during endoscopic retrograde cholangiopancreatography (ERCP), with roughly one-half of events occurring more than 24 hours after the procedure.^1^, ^2^, ^3^ Although most bleeds are self-limited, 20% to 30% still demand urgent endoscopic therapy. In addition, current surveillance techniques—such as serial hemoglobin checks, risk scores, and repeat endoscopy—miss intermittent bleeding and add cost and increase morbidity.^4^, ^5^, ^6^

A wireless, single-use capsule (PillSense; EnteraSense, Galway, Ireland) approved by the U.S. Food and Drug Administration in 2023 detects upper gastrointestinal blood optically, with >90% reported sensitivity and specificity, yet its value for post-ERCP monitoring is unknown.^7^

We affixed this capsule to the duodenum of 5 high-risk patients undergoing ERCP to provide continuous, real-time intraluminal sensing for bleeding. This case series assesses feasibility, signal accuracy, and clinical impact, testing whether anchored capsule monitoring can enable earlier, less-invasive detection of delayed PPB and guide timely intervention.

## Methods

This case series included 5 consecutive patients undergoing ERCP with ES or ampullectomy, identified by the endoscopist as high-risk for PPB (Table 1). Selection followed predefined criteria, including procedure-related factors (complex ERCP with precut sphincterotomy, ampullectomy for lesions >2 cm, or intraprocedural bleeding requiring hemostasis) and patient-related factors such as end-stage renal disease, recent anticoagulant use, or coagulopathy (international normalized ratio >1.5 or platelets <50 k/μL). This risk-stratified approach aligns with American Society for Gastrointestinal Endoscopy guidelines.

**Table 1:** Baseline characteristics and outcomes of patients monitored for postprocedural bleeding

Patient	Age, years	Gender	Procedure	Capsule reading	Repeat endoscopy	Bleeding seen on endoscopy	Migration	Outcome
1	59	F	ERCP + ES	Negative	No	N/A	N/A	Discharged safely
2	72	M	ERCP + ampullectomy	Positive	Yes	Yes	Yes	Successful hemostasis
3	56	F	ERCP + NKS	Positive	Yes	Yes	Yes	Discharged safely
4	65	M	ERCP + ampullectomy	Negative	Yes	Yes	No	Successful hemostasis
5	78	F	ERCP + NKS	Positive	Yes	No	Yes	Discharged safely

*ERCP,* Endoscopic retrograde cholangiopancreatography; *ES*, endoscopic sphincterotomy; *F*, female; *M*, male; *N/A*, not available; *NKS*, needle-knife sphincterotomy.

**Figure 1:**
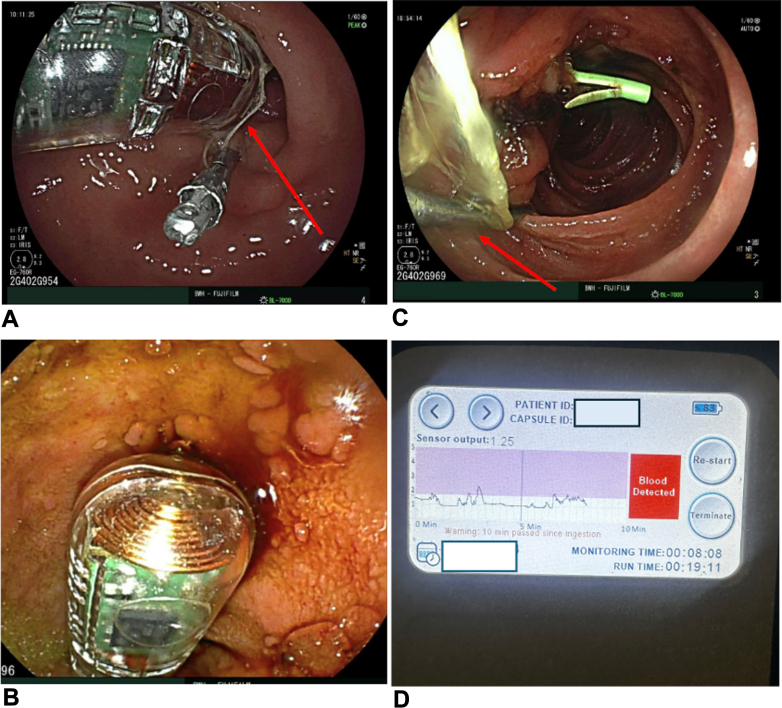
An implantable bleeding sensor for detection of postprocedural bleeding. **A,** A 2.0 VICRYL suture (Ethicon, Somerville, NJ, USA) was passed through the eyehole of the PillSense capsule (EnteraSense, Galway, Ireland) and tied in a knot (*arrow*). **B,** The capsule was affixed to the duodenal wall adjacent to the procedural site using an endoscopic clip (*arrow*). **C,** The capsule is seen in a pool of blood in the duodenum 1 day after placement. **D,** The bleeding sensor monitor indicates the detection of blood.

Patients met standard ERCP indications without contraindications to capsule monitoring and were observed for 24 to 48 hours using a bleeding sensor affixed to the duodenal wall. The capsule (PillSense) was prepared with a 2.0 VICRYL suture (Ethicon, Somerville, NJ, USA) tied in a knot through the capsule eyehole (Fig. 1A), then front-loaded onto a standard endoscope using a capsule delivery device (AdvanCE Endoscope Delivery Device; STERIS, Dublin, Ireland). An 11-mm endoclip (Resolution 360; Boston Scientific, Marlborough, Mass, USA) was placed around the suture loop to anchor the sensor near the procedural site in the duodenal bulb (Fig. 1B). If blood was detected (Fig. 1C), the sensor output signaled a positive reading (Fig. 1D).

## Case 1

A 59-year-old woman with acute myelogenous leukemia presented with right upper quadrant tenderness. Ultrasound and magnetic resonance cholangiopancreatography revealed common bile duct (CBD) dilation and a filling defect consistent with a stone. She initially underwent ERCP with biliary stent placement because of thrombocytopenia but was readmitted 3 months later with ascending cholangitis from stent occlusion, requiring repeat ERCP. Preprocedural hemoglobin was 8.9 g/dL (reference range: 12.0-15.5 g/dL). She was not on anticoagulation.

After removal of the occluded stent, an 8-mm CBD stone was extracted and ES performed. Moderate bleeding resolved with balloon tamponade. The bleeding sensor was deployed and secured 2 cm proximal to the sphincterotomy to avoid interference. The initial capsule reading was positive, prompting extended observation. After 8 hours of stable monitoring and a negative reading at 6 hours, she was discharged the same evening. At 3-month follow-up, hemoglobin remained stable and computed tomography (CT) confirmed resolution of biliary dilation.

## Case 2

A 72-year-old man with hypertension and prediabetes was referred for resection of periampullary tubulovillous adenoma with high-grade dysplasia. Endoscopic ultrasound showed no intraductal extension. ERCP with ampullectomy of the 50-mm × 30-mm lesion was performed. Preprocedural hemoglobin was 14.4 g/dL (reference range: 13.5-17.5 g/dL). The patient was not on anticoagulation. ES was performed using a monofilament traction sphincterotome. The bleeding sensor was secured into the duodenal bulb.

The sensor was positive at 12 hours despite the patient being asymptomatic with stable vital signs. This prompted urgent laboratory evaluation several hours before scheduled protocol that showed hemoglobin decrease to 12.8 g/dL. Repeat endoscopy revealed hematin in the gastric body and with 2 oozing vessels at the ampullectomy site (Forrest IIA/IIB). The sensor had migrated. Hemostasis was achieved with coagulation graspers and hemostatic gel. He remained stable and was discharged the next day. Hemoglobin remained stable over 4-month follow-up.

## Case 3

A 56-year-old woman with metastatic breast cancer presented with elevated liver function tests and dark urine. CT scan revealed a malignant CBD stricture at the pancreatic head. ERCP with needle-knife ES was unsuccessful, and no intraprocedural bleeding was noted. However, within hours, she developed melena with hemoglobin decreasing from 9.1 to 7.9 g/dL (reference range: 12.0-15.5 g/dL) and blood urea nitrogen increasing from 6 to 11 mg/dL (reference range: 7-20 mg/dL). She was not on anticoagulation.

Repeat ERCP the next day achieved biliary access, and a covered metal stent was placed. Although no active bleeding was seen, a clot and ulceration at the major papilla suggested recent bleeding. A bleeding sensor was placed in the duodenal bulb.

Two days later, the sensor returned a positive reading, and hemoglobin decreased from 10.6 to 8.7 g/dL, prompting a third endoscopy. No active bleeding was seen, but friable mucosa with a clean-based ulcer suggested intermittent bleeding. The sensor migrated to the distal small bowel during the procedure and was not retrieved. The patient remained stable without adverse events during follow-up.

## Case 4

A 65-year-old man with a history of myocardial infarction (age 40 years), atrial fibrillation on chronic anticoagulation, multiple myeloma in remission on thalidomide, and end-stage renal disease requiring hemodialysis presented with nausea and vomiting. Imaging revealed choledocholithiasis and cholelithiasis without cholecystitis. He underwent ERCP with ES and stone retrieval at an outside hospital, where an incidental ampullary adenoma was found. Preprocedural hemoglobin was 10.3 g/dL (reference range: 13.5-17.5 g/dL).

He subsequently underwent ERCP with piecemeal ampullectomy of the 30-mm lesion using snare electrocautery. Because of the high risk for PPB, a bleeding sensor was clipped into the duodenal bulb.

On postoperative day 1, he developed hematochezia, with hemoglobin decreasing to 8.7 g/dL and blood urea nitrogen increasing to 60 mg/dL. The sensor remained negative. Urgent endoscopy revealed a cratered ulcer with an adherent clot at the major papilla, which began oozing after suction; the sensor was visualized in place. Hemostasis was achieved with a 17-mm hemostatic clip, and hemoglobin stabilized after 1 unit of packed red blood cells. Anticoagulation was restarted on postoperative day 5, and no recurrent bleeding occurred during 4 months of follow-up.

## Case 5

A 78-year-old woman with a history of invasive bladder cancer, cervical cancer, and impaired renal function presented with acute abdominal pain. Liver function test results were elevated, and CT showed marked biliary dilation, confirmed on magnetic resonance cholangiopancreatography. She was not on anticoagulation, and pre-ERCP hemoglobin was 9.6 g/dL (reference range: 12.0-15.5 g/dL).

A plastic stent was placed in the ventral pancreatic duct. After unsuccessful biliary cannulation, a needle-knife ES was performed. Bleeding occurred from the sphincterotomy site and was managed with coagulation, achieving partial hemostasis within 3 minutes, although mild persistent oozing remained. A bleeding sensor was placed in the duodenal bulb for monitoring.

The next day, the sensor returned a positive reading with hemoglobin dropping to 7.4 g/dL. Repeat endoscopy showed no active bleeding, although clean-based ulceration was seen at the site of needle-knife ES. The sensor was no longer visible, suggesting distal migration. No recurrent bleeding or adverse events occurred during follow-up.

## Discussion

This case series assessed the feasibility of a capsule-based sensor that was fixed in the gastrointestinal lumen for detecting PPB after ERCP with ES or ampullectomy (Table 1). The sensor identified intraluminal blood in 3 patients, with 2 true positives requiring timely hemostasis. One false-positive result occurred, and another patient with an initial negative reading later had bleeding confirmed endoscopically. One true-negative result supported safe discharge.

Compared with traditional approaches, the capsule offers simple, continuous, real-time noninvasive detection without requiring serial laboratory tests or repeat endoscopy. By identifying subtle or intermittent bleeding, it may enable earlier intervention and reduce prolonged or additional hospital admissions, as in the patient in case 2, in whom delayed bleeding was identified and intervened upon before clinical deterioration. Conversely, case 1 highlighted how persistent negative readings after a high-risk procedure and an initial alert supported safe discharge. Because delayed PPB is difficult to predict, often occurs after discharge, and carries a greater rate of morbidity,^8^ continuous monitoring could complement existing tools, providing a safer option for high-risk patients while minimizing unnecessary intervention for low-risk patients.

Sensor migration occurred in 3 cases. Factors such as attachment technique, clips used for attachment, and the suture used for attachment may have contributed. Greater-strength clips, over-the-scope devices, reinforced sutures, or other attachment techniques may improve stability. Migration did not cause adverse events, although it could contribute false signals and diagnostic uncertainty, as seen in the patient in case 5, in whom the capsule read positive for bleeding, but no bleeding was seen at the procedural site (Table 1).

This study is limited by small sample size, restricting generalizability and precluding definitive conclusions. Variability in placement, orientation, and attachment may have contributed to detection failures, as in the patient in case 4, in whom bleeding was missed despite a visible ulcer. Larger studies are needed to validate performance and optimize deployment techniques.

## Conclusions

This novel bleeding sensor shows promise as a proof-of-concept adjunct for real-time PPB detection, potentially reducing unnecessary endoscopies and hospital stays. However, technical and migration challenges highlight the need for improved attachment strategies. It should be viewed as a complementary tool rather than a replacement for standard monitoring.

## Patient Consent

The patients in this article have given written informed consent to publication of their case details.

## Disclosure

The following authors disclosed financial relationships: S. Steinway: Consultant for Microvitality, Inc. All other authors disclosed no financial relationships.
